# Provincial trends in Legionnaires’ disease are not explained by population structure in Denmark, 2015 to 2018

**DOI:** 10.2807/1560-7917.ES.2021.26.25.2000036

**Published:** 2021-06-24

**Authors:** Kelsie Cassell, Daniel Thomas-Lopez, Charlotte Kjelsø, Søren Uldum

**Affiliations:** 1 Department of Epidemiology of Microbial Diseases, Yale School of Public Health, New Haven, United States; 2 These authors contributed equally to the work; 3 European Public Health Microbiology Training Programme (EUPHEM), European Centre for Disease Prevention and Control (ECDC), Stockholm, Sweden; 4 Department of Bacteria, Parasites and Fungi, Infectious Disease Preparedness, Statens Serum Institut, Copenhagen, Denmark; 5 Department of Infectious Disease Epidemiology and Prevention, Statens Serum Institut, Copenhagen, Denmark

**Keywords:** Legionella, incidence, Denmark, Legionnaires' disease, temporal trends, non-outbreak

## Abstract

**Background:**

Legionnaires’ disease (LD) incidence has been increasing in several European countries since 2011. Currently, Denmark is experiencing one of the highest annual incidences of LD despite its relatively cold climate and homogenous population, and the incidence differs notably across the country.

**Aim:**

We sought to determine whether provincial differences in LD incidence are attributable to the age and sex distribution of the population, and to characterise the risk of LD by province and age group in Denmark.

**Methods:**

Using national routine surveillance data for domestic LD cases collected between 2015 and 2018, we assessed the incidence of disease by province and year. Poisson regression models were fit to understand the risk of LD by year and province, as well as by 5-year age groups.

**Results:**

Incidence of domestic LD increased 48% between 2015 and 2018 across Denmark. Some provinces continuously had a high incidence of disease, even after adjusting for yearly trends and the underlying population distribution. Variations in the proportion of the population aged 65 years and older were not responsible for the increase in disease in our analysis. Finally, incidence of disease increased with each 5-year age group in both men and women.

**Conclusions:**

The relative differences in incidence between Danish provinces could not be explained by the age and sex distribution of the population, indicating that other factors must be responsible for the varying incidence across the country. These results may help inform trends in other countries in Europe also experiencing an unexplained high incidence of LD.

## Introduction

*Legionella* spp. are waterborne bacteria that can cause severe pneumonia and are transmitted through aerosols from sources such as showers, fountains, mist machines, spa pools, and cooling towers [1]. The incubation period for Legionnaires’ disease (LD) typically ranges between two and 10 days and common symptoms include fever, respiratory problems (cough, dyspnoea) and headaches [2]. Disease risk increases with age and men have a greater risk than women [3,4]. Certain comorbidities are also associated with disease, such as chronic obstructive pulmonary disease, diabetes, and immunosuppressive treatment [2,5].

LD is primarily caused by *Legionella pneumophila* in European countries [6]. The notification rate for LD has increased in the European Union/European Economic Area (EU/EEA) from 1.2 in 2011 to 1.8 cases per 100,000 inhabitants in 2018 and, from 2016 to 2017 alone, there was a 30% increase in reported cases [7,8].

The drivers for the general increase in LD across Europe are unknown but possibly include elevated testing intensity rates, fluctuations in weather conditions that facilitate the growth and spread of the bacteria, and changes in population structures as average age increases [9-11]. In addition to the general increase in LD, there is also considerable variability in incidence between countries, which could possibly be explained by variations in diagnostic test use (PCR, culture, or urine antigen test), efficiency of the surveillance systems, colonisation rates of water systems, climate and/or geographically-linked risk factors such as distance to cooling towers, and other water systems dispersing an aerosol to the environment [12-14].

Since 2013, Denmark has had one of the highest LD incidence rates of all countries reporting to the European Centre for Disease Prevention and Control (ECDC). Additionally, Denmark has shown an increasing annual incidence of disease, which reflects trends noted in other EU countries [8]. Within the country, incidence is not uniform, and some provinces have relatively high incidence despite no known outbreaks. For these reasons, we conducted the present study to investigate the underlying reasons for the increase in LD in Denmark and the distribution of cases by province. We estimated the relative risk (RR) for disease associated with increasing age in 5-year groups as well as the RR associated with residence in the different Danish provinces. We also examined the interaction between population and time to determine whether the increase in disease is due to an increase among the population of high risk, elderly people.

## Methods

### Setting, surveillance and data collection 

This is an observational study of all confirmed and probable cases of LD collected through a mandatory reporting system coordinated by the Danish national surveillance system. Denmark is a small country with a population of 5.8 million people. It is comprised of Jutland, the mainland area connected to the north of Germany, and a series of islands between Jutland and Sweden, which includes Zealand with the capital, Copenhagen (Supplementary Figure S1). There are 11 provinces, with the most populous being Copenhagen city. For this study, only domestic cases (including domestic travel) acquired in Denmark were analysed. Cases occurring in Bornholm province were excluded because of its distance from the rest of the country and extremely small population size (n = ca 39,000). 

The study period was limited to 2015–18 because no changes were made to the national surveillance system for LD during this time and because this period included the increase in LD cases that occurred in 2017 and 2018. As part of mandatory national surveillance, physicians are required to report confirmed or probable LD cases. The Department of Infectious Disease Epidemiology and Prevention at Statens Serum Institut in Denmark, together with the Danish Patient Safety Authority, collects data on symptom onset, hospitalisation, travel history, and demographic characteristics through case interviews and medical chart reviews.

Case data used for this particular study included age group, sex, travel history, province of residence, confirmation status of disease, and year of symptom onset (or hospitalisation date if onset date was unknown). Cases were aggregated into age groups of 5-year increments between the ages 0–95 years. The final age groups were: 0–19, 20–24, 25–29, 30–34, 35–39, 40–44, 45–49, 50–54, 55–59, 60–64, 65–69, 70–74, 75–79, 80–84, 85–89, 90–94, 95–119. The analyses were performed at the province (“Landsdele”) level in Denmark and provincial age- and sex-specific population data for years 2015–18 were obtained from Statistics Denmark, using the population numbers on the first day of the third quartile of each year [15].

### Case definition

Both ‘confirmed’ and ‘probable’ LD cases were considered in this analysis. Denmark uses the EU clinical case definition criteria for classifying confirmed and probable LD cases [16]. A ‘confirmed’ case is a pneumonia case confirmed by culture, urinary antigen test, or a four-fold rise in *L. pneumophila* serogroup 1 (*Lp* SG1) antibodies in paired serum samples. A ’probable’ case is a pneumonia case diagnosed by PCR, serology (paired antibody titre rise to other *L. pneumophila* serogroups or other *Legionella* spp.) or single positive antibody titre result to *Lp* SG1. 

Culture and PCR were the most commonly used diagnostic techniques during the study period from 2015 to 2018. In 2017 and 2018, 48% and 34% of the LD cases were verified via culture, respectively. The proportion of patients who were PCR-positive remained relatively consistent, constituting 81% in 2017 and 78% in 2018. Of note, patients can be positive by more than one method [17,18].

### Statistical Analysis

To estimate the RR of LD in each province and RR associated with year, Poisson regressions were fit using data aggregated by province and year. The unadjusted model estimated incidence with a covariate for each province. The fully adjusted model included covariates for province, year, population in each age group and sex without interactions. This model did not include an interaction between year and province because there was not a robust interaction between year and province in our analysis (p > 0.05 for all year and province combinations) and excluding this interaction improved the overall fit of our model. A separate model was used to examine the interaction between the population 65 years and older and year to determine whether an increase in population of the higher risk age groups was driving the increase in LD. All Poisson models included an offset for the natural log of the population.

In order to evaluate whether the increase in LD in Denmark and the distribution of cases by province were due to its population structure, we estimated age- and sex-standardised incidence rates for Denmark’s provinces from 2015–18. Provincial incidence rates (IR) were standardised using Poisson regression adjusting for differing sex and age population distributions of the provinces. The resulting regression coefficients were used to estimate the number of cases occurring in each age, sex, and province grouping for each year in Denmark, which was then used to create standardised IR. Covariates included year (as categorical), province, and sex. Population size was included as an offset term. In order to perform this, a reference age, sex, and province was necessary. The reference group for estimated standardized incidences of age, sex, and province for our study was men, ages 50–54, residing in Copenhagen city.

Finally, to estimate the RR of LD associated with increasing age, a Poisson regression was fit to the national LD data aggregated by age group (ranging from 20 and 119 years, with ages 85 years and above collapsed into a single category), adjusting for year (as a categorical covariate). This analysis was also performed for men and women separately where the reference group was men aged 50–54.

Data were extracted, merged, analysed and compared using Stata (version 14, StataCorp, College Station, Texas, United States (US)), Microsoft Excel 2013, PowerPivot for Excel and R statistical software version 3.4.3 (R Foundation, Vienna, Austria; packages: “lubridate”, “ggplot2”, “foreign”, “multcomp”).

### Ethical Statement

In accordance with Danish law, no informed consent was required for this study because our national epidemiological monitoring uses anonymised routine surveillance data. The planning and execution of this study is in line with the Declaration of Helsinki, as revised in 2013 [19].

## Results

From 2015–18, there were 651 confirmed or probable domestic cases of LD in Denmark (excluding three cases in Bornholm). LD cases peaked in 2017 with 202 cases and fell slightly to 194 cases in 2018, which was a 48% increase from the 131 cases occurring in 2015. PCR was the sole detection method for 28% (182/651) of cases and 45% (296/651) were confirmed via culture; the remaining cases were detected through the urine antigen test. Of the total cases, 37% (238/651) were women, which was consistent throughout the study period (ranging from 34% (66/194) in 2018 to 38% (77/202) in 2017). The RR of LD associated with year was higher in both 2017 and 2018 compared with 2015, but not in 2016 (Table 1).

**Table 1:** Unadjusted and adjusted relative risk of Legionnaires’ disease by province and year in Denmark, 2015–2018

Province	Cases	Population^a^	Crude RR	95% CI	Year, age, and sex-adjusted RR	95%CI
**Copenhagen city**	41	777,218	Reference		Reference	
**Copenhagen surrounding area**	47	546,013	1.61	1.06–2.45	1.15	0.76–1.75
**East Jutland**	116	885,515	2.47	1.73–3.52	1.82	1.27–2.60
**East Zealand**	36	248,583	2.72	1.74–4.25	1.86	1.19–2.91
**Funen**	122	497,134	4.59	3.22–6.54	2.96	2.08–4.22
**North Jutland**	36	588,888	1.14	0.73–1.78	0.73	0.47–1.14
**North Zealand**	62	462,938	2.50	1.69–3.72	1.55	1.05–2.23
**South Jutland**	93	725,236	2.39	1.65–3.45	1.54	1.07–2.23
**West and South Zealand**	67	587,796	2.13	1.44–3.13	1.28	0.86–1.88
**West Jutland**	31	430,853	1.34	0.84–2.14	0.87	0.55–1.39
**Year**						
**2015**	131	5,638,415	NA		Reference	
**2016**	124	5,684,627	NA		0.93	0.73–1.19
**2017**	202	5,720,890	NA		1.48	1.19–1.85
**2018**	194	5,750,174	NA		1.40	1.12–1.75

CI: Confidence interval; NA: Not applicable; RR: Relative risk.

^a^ Population data listed for individual provinces are based on 2018.

The analysis excludes the island province of Bornholm.

### Province-level risk

**Figure 1:**
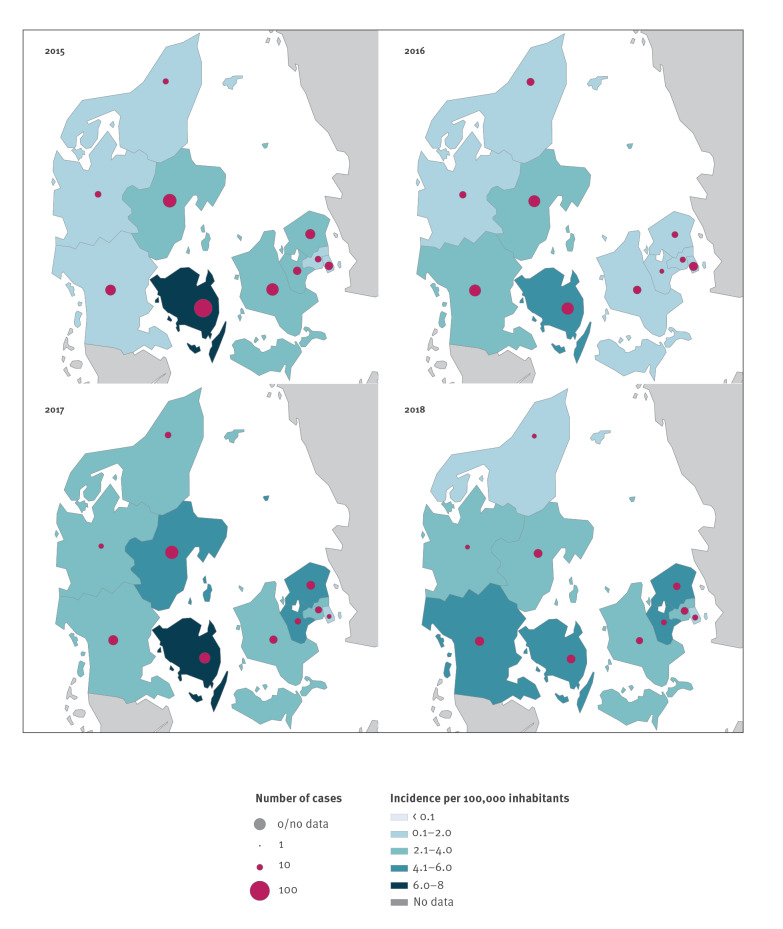
Map of domestic Legionnaires’ disease incidence by province and year, Denmark, 2015–2018 (n = 651)

**Figure 2:**
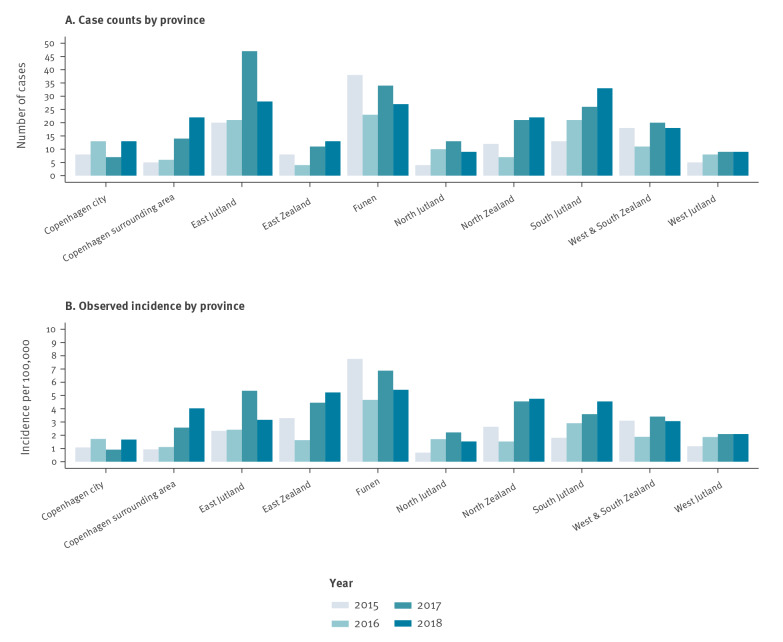
Cases and crude incidence rate of domestic Legionnaires’ disease by province, Denmark, 2015–2018

Nationwide trends in domestic incidence were not uniform across all 10 Danish provinces included in the analysis (Figure 1). The Copenhagen surrounding area and South Jutland showed a steady increase in cases throughout the study period, while Funen consistently had the highest incidence of LD each year. East Jutland experienced a peak in cases during 2017 with a lower incidence both before and after 2017 (Figure 1 and 2). In contrast, West Jutland and Copenhagen city had a relatively stable incidence of disease during the study period. 

Incidence is per 100,000 inhabitants based on the annual province population.

Incidence is per 100,000 inhabitants based on the annual province population.

Cases and crude incidence rate of LD varied substantially across provinces during the study period. Between 2015 and 2018, cases reported in Copenhagen city ranged between only seven and 13 per year (Figure 2). North Jutland, Copenhagen city, and the Copenhagen surrounding area experienced less than 1 case per 100,000 inhabitants in 2015, 2017, and 2015, respectively. The highest incidence in Denmark during the study period was 7.8 cases per 100,000 inhabitants in Funen during 2015. This could potentially be attributed, in part, to a suspected outbreak (all serogroup 1 Knoxville sequence type 9) affecting between nine and 15 people in Odense, the main city of Funen. Removing 15 cases yields an incidence rate of 4.7 per 100,000 inhabitants, which is still the highest incidence rate among all provinces in 2015. Incidence rates of 6.9 cases per 100,000 inhabitants in Funen in 2017 and 5.4 cases per 100,000 inhabitants in East Jutland in 2017 were the next highest rates observed. 

**Figure 3:**
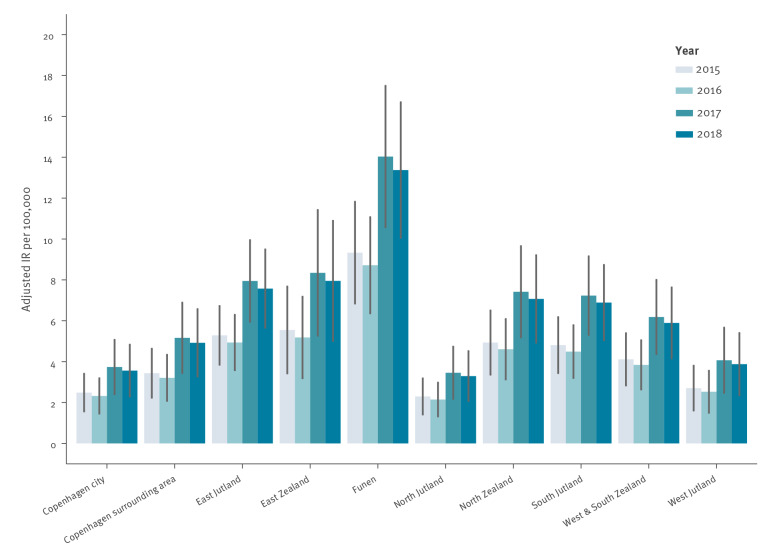
Age- and sex-adjusted Legionnaires’ disease incidence rate by province and year, Denmark, 2015–2018

Using Copenhagen city as reference, all provinces studied had a greater unadjusted RR of LD with the exception of North Jutland and West Jutland (Table 1). After adjustment for year, age, and sex, the residents of the Copenhagen surrounding area, in addition to North Jutland and West Jutland, had a lower estimated risk of reported LD compared with residents of Copenhagen city, although the effect was not robust (RR: 1.15, 95% CI: 0.76–1.75). The crude RR of reported domestic LD in Funen (RR: 4.59, 95% confidence interval (CI): 3.22–6.54) was almost four times greater than that of Copenhagen city during the study period (Table 1). Although this RR decreased after adjusting for yearly trends as well as age and sex distributions of the underlying population, the age and sex standardisations did not change the overall relative trends in incidence by province or year. Indeed, five provinces had a higher RR than Copenhagen city (Figure 3). It is important to note that the standardised IR shown in Figure 3 cannot be directly compared with the observed IR shown in Figure 2 as the standardisation process assumes a different underlying population structure (reference group: 50–54-year-old men), which is a higher risk age and sex group and hence yields the higher estimated IR in Figure 3. There were no robust interactions between year and province indicating that the yearly fluctuations during this period were not substantial when considering each province individually. Thus, an interaction term for year was not included in the final adjusted analysis presented.

Between 2015 and 2018, the population of people aged 65 years and older increased 6% among the 10 Danish provinces studied. As expected, an increase in this age group within the population was an independent risk factor for disease. However, there was no interaction between the population 65 years and older and year in our regression models, indicating that the effect of an older population was not modified by specific years.

### Age-associated risk

In total, 68% (444/651) of domestic cases were among individuals between ages 50 and 79 years old. Between the ages of 50 and 89, women consistently made up ca 30–40% (16/53–28/71) of reported cases. The average annual incidence rate during the entire study period by age group ranged from 0.19 (among 20–24-year-olds) to 18.44 cases (among 90–94-year-olds) per 100,000 age-specific inhabitants (Supplementary Figure S2). The greatest number of cases occurred among the 70–74- and 75–79-year-old age groups, while the largest age-specific IR was recorded in the 85–89-, 90–94-, and 95–119-year-old age groups. For some age groups (30–34, 50–54, 70–74, and 90–94), incidence has generally increased each year, as exemplified by the observation that 70–74-year-olds had an IR of 6 cases per 100,000 inhabitants (age-specific population) in 2015, which increased to 11 cases per 100,000 inhabitants (age-specific population) in 2018.

**Figure 4:**
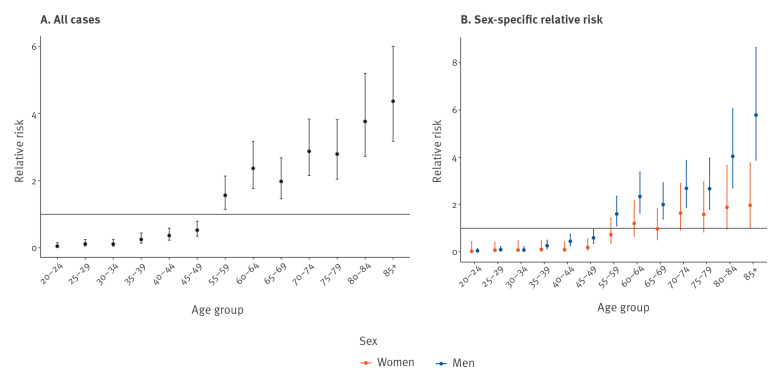
Relative risk of Legionnaires’ disease by 5-year age category, Denmark, 2015–2018

The RR of LD was 2.46 (95% CI: 1.70–3.58) times greater among 60–64-year-olds compared with 50–54-year-olds (Figure 4). The risk continued to increase with age and 80–84-year-olds had 5.55 (95% CI: 3.78–8.15) times the risk and 90–94-year-olds had 7.46 (95% CI: 4.54–12.26) times the risk of reported disease compared with the 50–54-year-old reference group. Both men and women had similar trends in RR of LD with age, however there was more variability in the estimates for women.

The reference group was (A) age group 50–54 years and (B) men in the age group 50–54 years.

## Discussion

Surveillance for LD is crucial to understanding the temporal and geographical trends of the disease. Our report demonstrates that provincial level data over a 4-year period in Denmark can provide important insights into domestic disease trends, which may be parallel data from other European nations.

During the study period, the observed annual domestic incidence rate in each province ranged from 0.7 (North Jutland in 2015) to 7.8 (Funen in 2015) cases per 100,000 inhabitants. The age- and sex-standardised annual incidence reflected similar provincial trends, as was noted among the crude annual incidences. This indicates that differences between provinces are probably not the result of the age and sex distributions of the populations in each province. Additionally, there was no significant statistical interaction between time and population 65 years of age and above, signifying a lack of evidence that the increase in cases over time can be attributed to a changing age structure within the population. This supports the findings published in the ECDC 2017 Annual Epidemiologic Report on Legionnaires’ disease that factors other than demographic changes might be responsible for the increasing notification rate in the EU/EEA countries, including changes in diagnostic tests, intensity of testing, weather and climate [8].

Between 2016 and 2017, most provinces in Denmark experienced an unexplained increase in LD, which remained high in 2018 as well. This sudden increase is not connected to any known outbreak. It is possible that the increase in LD observed in 2017 follows a general trend that began in 2011. Thus, although a relatively large leap in cases was observed between 2016 and 2017, this could reflect an unusually low number of cases in 2016, which may be an outlier in a series of years showing an increasing trend in disease. These findings warrant additional thorough investigations into how established risk factors like rainfall, humidity, temperature, and climate influence the yearly fluctuations of LD in Denmark [11]. LD is sometimes casually referred to as a Mediterranean disease as countries with warmer annual climates such as Italy and Spain historically report high incidence rates. However, Denmark has a starkly different climate compared with these countries [8,20]. Denmark also employs multiple methods of LD identification (culture and PCR in addition to urine antigen test). In addition, it is possible that physicians in Denmark have a different level of awareness, which could partially explain why the country has on average a higher incidence compared with some other European countries. This does not fully explain why there has been an increase and why certain provinces have such differing trends in disease.

Reports from other locations including the UK, the state of Connecticut or New York City in the US show a high incidence of LD in urban, populous areas [21,22]. Typically, the higher incidences of LD found in cities are attributed to large buildings, plumbing systems, cooling towers and other environmental sources related to urban infrastructure. In addition, larger hospital systems in urban areas may have better diagnostic capacity and testing practices [10,23]. This is in contrast to the situation in Denmark, which is a small country in both land area and population, and is considered homogeneous in many regards [24,25]. Funen is home to Odense, the third largest city in Denmark, but is the seventh-smallest province by population and the sixth-smallest by population density. Despite its relatively low population density, Funen consistently had the highest incidence of LD during the study period. While adjustment for population distribution reduced the RR estimates, no provinces had a significantly lower RR for LD than Copenhagen city. Copenhagen city’s population is skewed on the young side: 60% of the population during the study period was less than 40 years of age, compared with 46% in the rest of Denmark. However, our analysis indicates that Copenhagen’s relatively low IR is not driven by the younger population, nor the underlying sex distribution.

Our report presents analyses describing the RR of LD associated with 5-year age groups. The age groups 70–74 and 85 years and older showed large increases in domestic LD incidence in 2017 and 2018 compared with 2015 and 2016 rates, but the reasons for this are unclear. The proportion of cases that were women was elevated among the older age groups, reflecting the underlying sex distribution among the older adults. Using more granular age group bands of 5 years, this analysis confirmed previous research identifying an association between age and RR for disease [26]. Future analyses should be focused on determining to what extent the age-dependent risk can be confounded by underlying comorbidities associated with age.

Our analyses were subject to certain limitations. The data analysed were limited to a short study period, between 2015 and 2018, when LD surveillance in Denmark was consistent. We were also not able to assess other potential factors that contribute to the increase in LD incidence during this time period, such as inclination to test and testing intensity. A physician’s inclination to test for and thus detect LD may also be dependent on the patient’s age, which we were unable to account for in this analysis. Testing intensity fluctuations could also contribute to the increase in reported LD and provincial differences in incidence. Assessments of this relationship and its impact on Danish LD incidence reports are currently in progress.

The proportion of cases associated with travel (excluded from this study) did not substantially change between 2015 to 2018, ranging from 26% (44/168) to 28% (51/182), respectively (Supplementary Figure S3). As the trend of travel-associated cases is similar to domestic cases, we can discard the idea that the increase in LD in Denmark can be explained by an increase in travel patterns. Additionally, our results indicate that certain regions are more likely to report domestic LD cases, but this does not provide information on whether people in certain provinces are at greater risk for acquiring the disease or being exposed to the disease.

## Conclusions

Our results reveal a lack of evidence that the increase in LD between 2015 and 2018 in Denmark is due to an ageing population. We have found large variations in incidence of disease by province that, while fluctuating over a short time period, reveal distinct patterns unexplained by the underlying population structures. This ultimately emphasises the need for regularly conducted regional analyses in order to adequately characterise trends over time. These results and descriptive statistics can be used to better inform research directions, auditing of diagnostic testing practices and understanding of LD rates in European countries with a concern about the rising incidence of disease.

## Supplementary Data

1018000 Supplement
