# Supplementary material for: Provincial trends in Legionnaires’ disease are not explained by population structure in Denmark, 2015 to 2018
Source: Euro Surveill. 2021 Jun 24;26(25):2000036. doi: 10.2807/1560-7917.ES.2021.26.25.2000036 (PMC8229376; doi:10.2807/1560-7917.ES.2021.26.25.2000036)
Supplement: Supplement [file 20-00036_CASSELL_Supplement.pdf]

Provincial trends in Legionnaires' disease are not explained by population structure in Denmark

This supplementary material is hosted by *Eurosurveillance* as supporting information alongside the article *Provincial trends in Legionnaires' disease are not explained by population structure in Denmark*, on behalf of the authors, who remain responsible for the accuracy and appropriateness of the content. The same standards for ethics, copyright, attributions and permissions as for the article apply. Supplements are not edited by *Eurosurveillance* and the journal is not responsible for the maintenance of any links or email addresses provided therein.

SUPPLEMENTAL FIGURES

Supplemental Figure S1. Map of Danish Provinces.

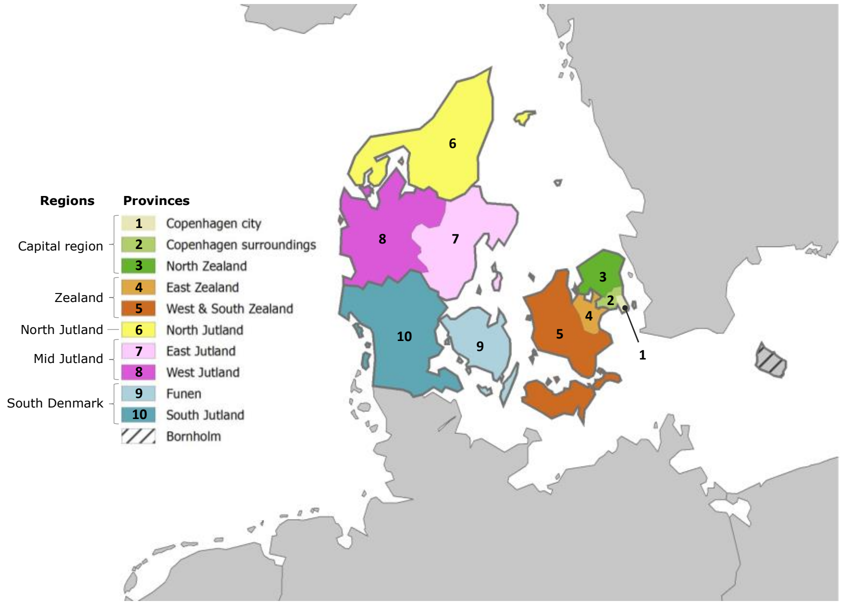

Supplemental Figure S2. Age-specific cases and incidence of Legionnaires' disease by year and sex, Denmark 2015-2018.

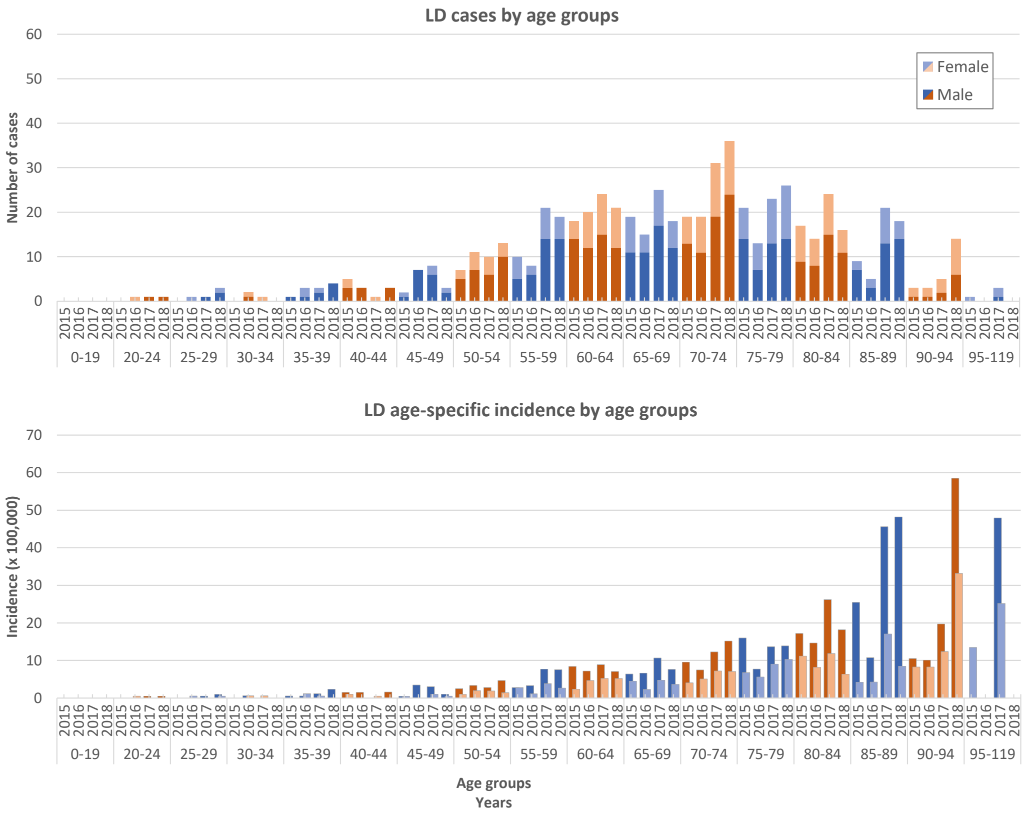

Supplemental Figure S3. Case counts and proportion of travel associated Legionnaires' disease cases by year, Denmark 2015-2018.

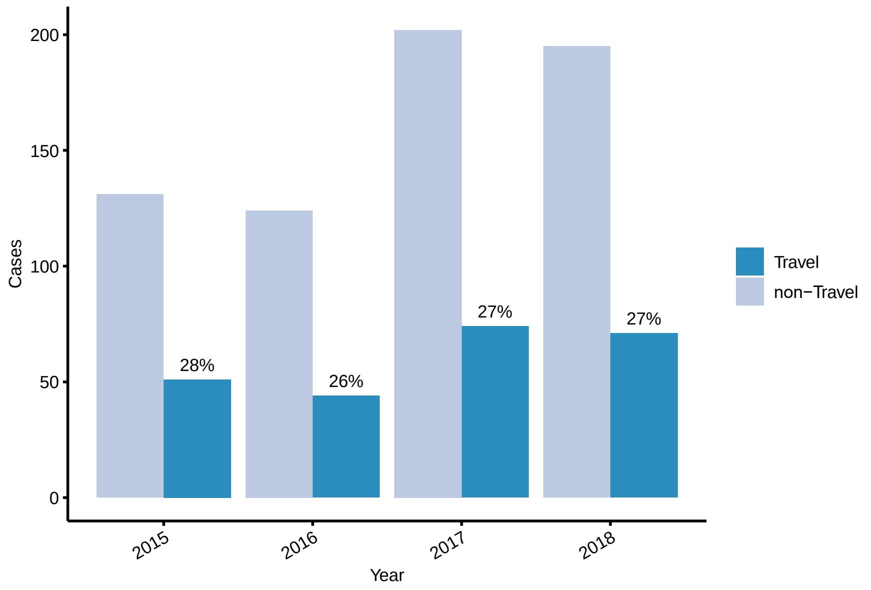

Note: the percentages above travel associated cases represent the percent of yearly LD cases that are travel associated.
